# Complete Genome Sequence of Lactobacillus fermentum Strain AGR1485, a Human Oral Isolate

**DOI:** 10.1128/MRA.00841-20

**Published:** 2020-09-03

**Authors:** Marc A. Bailie, Eric Altermann, Wayne Young, Nicole C. Roy, Warren C. McNabb

**Affiliations:** a School of Food and Advanced Technology, Massey University, Palmerston North, New Zealand; b Food Nutrition & Health Team, AgResearch, Grasslands Research Centre, Palmerston North, New Zealand; c Riddet Institute, Massey University, Palmerston North, New Zealand; d The High-Value Nutrition National Science Challenge, Auckland, New Zealand; e Department of Human Nutrition, University of Otago, Dunedin, New Zealand; f Liggins Institute, University of Auckland, Auckland, New Zealand; University of Rochester School of Medicine and Dentistry

## Abstract

Lactobacillus fermentum is found in food products and is generally considered safe. *L. fermentum* AGR1485 promotes barrier integrity in Caco-2 cells and has genetic similarities to other known probiotic *L. fermentum* strains. *L. fermentum* AGR1485 has potential as a probiotic and was sequenced to explore these probiotic properties. The genome is a 2.2-Mbp circular chromosome with no plasmids and a GC content of 51.15%.

## Abstract (precis)

Lactobacillus fermentum is found in food products and is generally considered safe. *L. fermentum* AGR1485 promotes barrier integrity in Caco-2 cells and has genetic similarities to other known probiotic *L. fermentum* strains. *L. fermentum* AGR1485 has potential as a probiotic and was sequenced to explore these probiotic properties. The genome is a 2.2-Mbp circular chromosome with no plasmids and a GC content of 51.

## ANNOUNCEMENT

Lactobacillus fermentum is commonly found in food products (1, 2) and is used as a probiotic therapeutic agent for the treatment of intestinal and vaginal pathogens (3–5). *L. fermentum* AGR1485 increases the barrier integrity of Caco-2 monolayers but does not induce colonic inflammation in germfree mice (6, 7). *L. fermentum* AGR1485 has genetic similarities to other known probiotic *L. fermentum* strains, but little is known about the probiotic mechanisms of action of these strains. *L. fermentum* AGR1485 was isolated by oral swab from a healthy human volunteer and identified as a member of the species *L. fermentum* using 16S rRNA gene sequencing (8). The genome of *L. fermentum* AGR1485 was sequenced to determine its probiotic properties and genetic characteristics.

*L. fermentum* AGR1485 was cultured in de Man-Rogosa-Sharpe (MRS) broth (Merck Ltd., Auckland, New Zealand) and incubated overnight at 37°C. The cells were lysed by grinding under liquid nitrogen followed by the Qiagen bacterial lysis method (9). High-molecular-weight DNA was purified on Qiagen Genomic-tip/100G columns (Bio-Strategy, Auckland, New Zealand) following the manufacturer’s specifications (9).

Whole-genome sequencing was done using both Illumina and PacBio single-molecule real-time (SMRT) sequencing for hybrid assembly. Illumina library creation and sequencing were carried out by BGI (Beijing Genomic Institute, Shenzhen, China), and PacBio SMRTbell library creation and sequencing were done by Novogene (Hong Kong, China). Illumina template DNA was sheared into 500-bp fragments for library creation using a TruSeq library preparation kit from Illumina. The resulting template DNA was sequenced on a HiSeq 2000 genome analyzer that generated 2,738,965 100-bp paired-end reads. The PacBio SMRTbell library was created from sheared template DNA, and the hairpin dimers were purified by magnetic beads with size selection conditions. The adapters were removed using PacBio’s MagBead kit. SMRT sequencing of the SMRTbell templates was carried out on a PacBio Sequel platform, creating 249,954 subreads with an average length of 8,790 bp and an *N*_50_ value of 10,057 bp.

Default parameters were applied for all software packages unless otherwise specified. Illumina reads were assessed for length, quality, and adapters before and after trimming using FastQC v0.11.9 (10). Illumina reads were quality controlled and trimmed with Trimmomatic v0.39 (11). Hybrid *de novo* assembly was performed with Unicycler v0.4.7 (12) using the trimmed Illumina short reads and uncorrected PacBio long reads. The assembly graph was checked for errors and completeness using Bandage v0.8.1 (13) before final assembly and polishing. Polishing was done with Pilon v1.22 (14) using all the sequencing reads until no further improvements to the assembly could be made.

**FIG 1:**
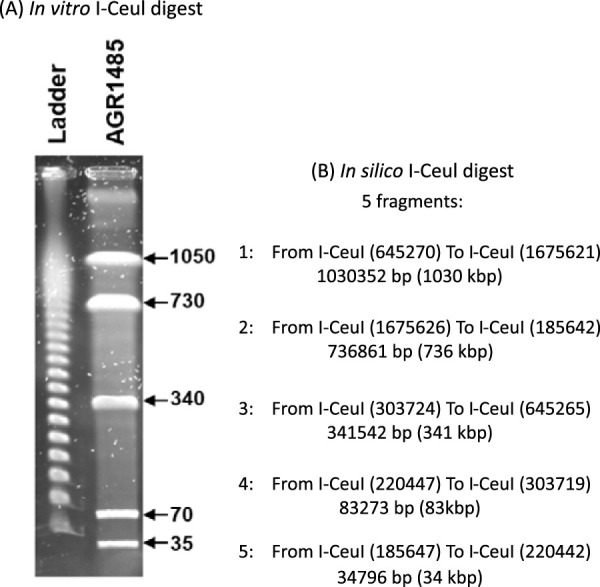
(A) Pulsed-field gel electrophoresis of AGR1485 genomic DNA digested with restriction enzyme I-Ceul. The marker ladder contained lambda DNA, where the fragments were multimers of 48.5 kb. The values given are the sizes (kb) of the DNA fragments from the bacterial strains. Image and caption adapted from Sengupta et al. (6) under a CC BY-NC 4.0 license. (B) *In silico* digest results of the AGR1485 genome assembly using I-Ceul restriction sites processed by UGENE (17) and presented as the range from one restriction site to the next (fragment sizes are in base pairs and kilobase pairs).

The polished assembly was assessed with CheckM v1.0.18 to have 99.18% completeness and 0.546% contamination (15). QUAST v4.6.3 (16) confirmed that the genome sequence was a single 2,226,862-bp chromosome with a GC content of 51.15% and contained no missing or ambiguous nucleotides. Read coverage for the assembly was calculated to be 989.2×. During assembly, Unicycler v0.4.7 predicted the chromosome to be circular. UGENE v34.0 (17) was used to overlap the ends of the *L. fermentum* AGR1485 sequence and calculate an *in silico* digest at the I-Ceul restriction sites. The *in silico* restriction digest fragment pattern and fragment sizes closely resembled previously published pulsed-field gel electrophoresis results of the same genome using a commercial I-Ceul restriction enzyme (Fig. 1) (6).

The assembly was annotated with PGAP v4.11 (18) and GAMOLA2 v16.0 (19), which found 2,367 open reading frames (ORFs), 1,839 Clusters of Orthologous Groups (COGs), and 1,920 conserved domains. The genetic characteristics that give rise to this organism’s unique phenotypes are likely harbored in this chromosome, because no plasmids were identified during DNA purification or assembly.

### Data availability.

The PacBio long reads (SRX7669246) and Illumina reads (SRX7669245) described here have been deposited at NCBI/GenBank under BioProject number PRJNA588334. The whole-genome sequence (accession number CP047584.1) is available from NCBI/GenBank under BioSample accession number SAMN13241836.
